# Induction of diapause and seasonal morphs in butterflies and other insects: knowns, unknowns and the challenge of integration

**DOI:** 10.1111/phen.12014

**Published:** 2013-05-06

**Authors:** Sören Nylin

**Affiliations:** Department of Zoology, Stockholm UniversityStockholm, Sweden

**Keywords:** Genetics, life history, photoperiod, plasticity, polyphenism, seasonality

## Abstract

The ‘choice’ of whether to enter diapause or to develop directly has profound effects on the life histories of insects, and may thus have cascading consequences such as seasonal morphs and other less obvious forms of seasonal plasticity. Present knowledge of the control of diapause and seasonal morphs at the physiological and molecular levels is briefly reviewed. Examples, mainly derived from personal research (primarily on butterflies), are given as a starting point with the aim of outlining areas of research that are still poorly understood. These include: the role of the direction of change in photoperiod; the role of factors such as temperature and diet in modifying the photoperiodic responses; and the role of sex, parental effects and sex linkage on photoperiodic control. More generally, there is still a limited understanding of how external cues and physiological pathways regulating various traits are interconnected via gene action to form a co-adapted complete phenotype that is adaptive in the wild despite environmental fluctuation and change.

## Introduction

How are organisms adapted to their local environment? This is a seemingly simple question, although it is in fact immensely complex because the full answer would span all the way from ecology and evolution to molecular processes, via physiological and neurobiological mechanisms that interconnect co-adapted traits. The study of adaptations to seasonality in insects may be one of the most promising areas of research that aims to answer this question because there is much ecological knowledge on insects under field conditions, as well as in *Drosophila melanogaster*, one of the best developed model systems for genetic and molecular studies. The challenge, however, is to integrate knowledge from these disparate fields to enable a real understanding of how an adapted phenotype is formed.

Local adaptations fit insects to the seasonal conditions where they are located, facilitating their survival and reproduction across an often dramatically wide range of environments. Arguably, the most important of these adaptations is diapause, the hormonally controlled physiological state of arrested development that is ubiquitous among insects and other arthropods in seasonal environments (Denlinger *et al*., 2011). This is because this major adaptation is so central in fitting the life cycle to local conditions. Also, in insects with facultative diapause, the ‘choice’ facing an individual insect (i.e. to enter diapause in preparation for a season not suitable for growth and reproduction or, instead, to develop directly to sexual maturation and produce an additional generation) is fundamental to the entire phenotype. From this choice, many other co-adapted traits follow, ranging from the often obvious seasonal morphs (i.e. forms differing between the two developmental pathways; Hartfelder & Emlen, 2011; Simpson *et al*., 2011) to more subtle but no less important plastic differences between pathways such as in life history traits (Nylin & Gotthard, 1998) or immunology (Lee *et al*., 2002; Prasai & Karlsson, 2011).

Here, the present knowledge of the control of diapause and seasonal morphs at the physiological and molecular levels is briefly reviewed. Examples, mainly derived from personal research (primarily on butterflies), are then given as a starting point with the aim of outlining areas of research where there is still a limited understanding of life cycle regulation, including the integration of co-adapted traits to ensure the formation of a complete phenotype that is adapted to local conditions. Such understanding is essential for predicting biological responses to changes in the environment.

## Diapause regulation

Subsequent to the pioneering efforts of Danilevskii (1965), the major external cue regulating diapause is known to be the photoperiod (often modified by temperature). Although there are exceptions, the typical pattern is that diapause is induced by a photoperiod with short days, interpreted by the insect as a late date when winter is approaching, although the critical photoperiod (where 50% of the individuals enter diapause) increases with latitude (Danilevskii, 1965; Tauber *et al*., 1986). This reflects local adaptation to the facts that winter comes earlier at high latitudes, and that summer day lengths are longer at a given date. Probably the best evidence of such local adaptation comes from the pitcher-plant mosquito *Wyeomyia smithii*, where latitudinal as well as altitudinal variation in photoperiodic thresholds for larval diapause fit theoretical expectations perfectly, demonstrating strong natural selection shaping the thresholds (Bradshaw, 1976). This system also provides the first and most powerful example of an evolutionary response to global warming, as a result of the demonstration that thresholds have shifted genetically towards shorter day lengths (Bradshaw & Holzapfel, 2001).

The genetics underlying the entry into diapause can be viewed as a set of interconnected ‘modules’, or ‘groups of functionally related genes that interact with each other to control integrated processes’ (Emerson *et al*., 2009). The photoperiodism module is composed of genes involved in: inputting the light signal, a timer, a counter, and an output signal to the next module, the hormonal events (genes regulating synthesis, secretion and degradation of hormones). Output from this endocrine system elicits the diapause module itself, which includes the genes behind not only cessation of development and reproduction, but also a whole syndrome of traits that are often associated with diapause (build-up of energy reserves, increased stress resistance, reduced metabolic rate, etc.). Genes belonging to a fourth module, the circadian clock, are also known to affect diapause induction via effects on any or all of the above modules, although Emerson *et al*. (2009) caution that individual clock genes can influence the photoperiodic response without the circadian clock being involved as a module *per se*. The precise role of clock genes for diapause needs further clarification (Saunders *et al*., 1989; Bradshaw & Holzapfel, 2010; Ikeno *et al*., 2010; Yamada & Yamamoto, 2011).

The state of present knowledge regarding the physiological and genetic regulation of diapause has been reviewed recently by Denlinger *et al*. (2011), and thus some of the patterns are only briefly summarized here to highlight differences and (in particular) similarities between systems. Diapauses in different developmental stages are distinguished because this appears to be the main divider among modes of regulation, rather than phylogeny.

### Embryonic diapause

The best studied insect regarding embryonic diapause is the silk moth *Bombyx mori*, although this species has an atypical diapause. Diapause in *B. mori* is under maternal control, and the stage that is sensitive to photoperiodic induction occurs so early in the mother's own life that the winter diapause is induced by long summer days and high temperature, rather than by short days and low temperature. The factor controlling this diapause is a hormone (simply called diapause hormone). The molecular processes behind this hormonal action are also relatively well known, with progress aided by the fact that the genome of *B. mori* is the most completely annotated Lepidoptera genome (Denlinger *et al*., 2011).

Embryonic diapause in other insects is regulated in diverse ways, although with more similarities to diapause in other developmental stages. As is also the case for larval and pupal diapause, a role for ecdysteroids is often implicated. This is the case, for example, not only in the gypsy moth *Lymantria dispar* (Lee & Denlinger, 1997), but also in several other moths and orthopterans (Denlinger *et al*., 2011). At the molecular level, several genes with unique diapause expression profiles have been identified, although most such differences between individuals in diapause or developing directly are likely to be downstream of the diapause induction itself. However, Reynolds & Hand (2009) find that several genes coding for proteins involved in ecdysteroid synthesis and signalling (CYP450, AKR and RACK1) are up-regulated in pre-diapause embryos of the cricket *Allonemobius socius* and down-regulated later in diapause.

### Larval and pupal diapause

Diapause in the larva or pupa are similar phenomena in that they both entail a failure to moult to the next stage of development. As noted by Denlinger *et al*. (2011), this in itself suggests the likelihood that the ecdysteroid hormones required to initiate the next moult are involved in diapause induction, and this has indeed been found to be the case. The classical model for induction of larval and pupal diapause involves an axis from the brain to the prothoracic gland, where a reduction in the production of prothoracicotropic hormone (PTTH) in turn reduces ecdysteroid secretion, thus preventing moulting. Several experiments showing high levels of dopamine in the brains of diapause-destined larvae or pupae (Noguchi & Hayakawa, 1997; Kostal *et al*., 1998) suggest that the initial prevention of release of PTTH is mediated by this neurotransmitter. Indeed, in the cabbage moth *Mamestra brassicae*, feeding l-3,4-dihydroxyphenylalanine (l-DOPA) to larvae reared in long day lengths to raise the levels of dopamine is enough to elicit a diapause-like state in more than 50% of individuals (Noguchi & Hayakawa, 1997).

There is much support for this classical view (Denlinger *et al*., 2011), and also support from gene expression studies. In the tobacco budworm *Heliothis virescens*, transcription of PTTH drops sharply before pupal diapause (Xu & Denlinger, 2003). With respect to the precise role of dopamine, studies on *M. brassicae* have begun to determine the molecular mechanisms of initial diapause induction. Uryu *et al*. (2003) searched for genes that were overexpressed in short day lengths or in larvae fed l-DOPA, and found that the most dramatically up-regulated gene under both condition is RACK, the receptor for activated protein kinase C. This family of protein kinase C genes transduces various signals involved in regulating cellular function, and it is thus likely that signal transduction through protein kinase C is involved in inducing diapause (Uryu *et al*., 2003).

The classical model does not appear to tell the whole story, however, because recent studies (Denlinger *et al*., 2011) have also indicated a role in larval and pupal diapause of diapause hormone and Juvenile Hormone (JH) for the maintenance and termination of diapause in some insects. Nevertheless, when it comes to the actual induction of diapause (the subject of the present review), the classical model remains well supported.

### Adult diapause

By contrast to larval and pupal diapause, adult diapause is not associated with a failure to moult but with delayed reproduction (reproductive diapause is sometimes used as a synonym). The classical model is that adult diapause is induced (and maintained) by a reduction in JH secretion from the corpora allata (CA), a pair of glandular organs located behind the brain. The activity of the CA is in turn controlled by the brain, both through hormones and neurones (Denlinger *et al*., 2011). One of the best studied systems is the mosquito *Culex pipiens*, where it is shown that CA dissected from diapausing females secrete very little JH (Readio *et al*., 1999).

Interestingly, at least in some species, the sexes differ in the scheme for the regulation of adult diapause. In the linden bug *Pyrrhocoris apterus*, the CA are involved in females but not in males (Hodkova, 1994); in the monarch butterfly *Danaus plexippus*, it appears that migrant males (in reproductive diapause) exhibit a down-regulation of JH synthesis, whereas females use an increased turnover and/or increased binding of JH to reduce the circulating levels (Zhan *et al*., 2011).

There is evidence that reduced insulin signalling is critical for induction of adult diapause (Tatar & Yin, 2001; Emerson *et al*., 2009). In *C. pipiens*, knockdown of the insulin receptor by RNA interference results in a diapause-like state of reproductive arrest, which can be terminated by the application of JH (Sim & Denlinger, 2008). *Drosophila melanogaster* is another species in which the insulin signalling pathway is strongly implicated in diapause regulation (Williams *et al*., 2006). In this and other species, there also appears to be a role for ecdysteroids with respect to inducing and maintaining adult diapause, and probably both types of hormone are important (Denlinger *et al*., 2011).

## Seasonal morphs and evolution

Insects often occur in distinct seasonal morphs; sometimes the morphs are tightly linked to diapause, sometimes less (Shapiro, 1976; Simpson *et al*., 2011). Such morphs are by their nature phylogenetically plastic, and the term seasonal polyphenism is used often to distinguish this plasticity from genetic polymorphism (Michener, 1961; Mayr, 1963). It should be noted, however, that plastic traits also have a genetic basis, and that there is often genetic variation in plasticity (Nylin & Gotthard, 1998; Brisson, 2010). Indeed, the distinction between morphs determined genetically or plastically is not nearly as strict as was previously assumed (West-Eberhard, 2003; Leimar, 2009) and a recent review of hormonal control of polyphenism by Hartfelder & Emlen (2011) also deals with cases of genetic polymorphism, including even sexual dimorphism. This makes sense if polymorphisms and polyphenisms are both seen as sets of alternative phenotypes, the development of which can be triggered by external or internal cues, or combinations of both (Leimar, 2009; Brisson, 2010).

The adaptive nature of seasonal morphs is sometimes obvious, as in the case of morphs associated with parthenogenetic or sexual reproductive modes in aphids (Simpson *et al*., 2011), or at least relatively clear, as for melanization in pierid butterflies and its role in temperature regulation. Evidence for the latter comes not only from functional studies (Watt, 1968; Kingsolver, 1987), but also from studies comparing insects from different seasons, altitudes and latitudes, showing many cases of parallel evolution of variation in melanization in relation to ambient temperatures (Shapiro, 1976, 1984). In other cases, the evidence is suggestive although not conclusive; one example is the seasonal forms of *Bicyclus* butterflies, where the more active wet season form has larger eyespots than the dry season form, presumably as a result of differences in selection from predators (Lyytinen *et al*., 2004). There are also cases where the adaptive function (if any) is something of a mystery, such as the conspicuous seasonal polyphenism of map butterflies *Araschnia levana* (Shapiro, 1976; Fric & Konvicka, 2002; Joiris *et al*., 2010). The possibility should not be discarded that polyphenism is not always adaptive *per se* but, in some cases, could just be a by-product of different developmental pathways (Gotthard & Nylin, 1995).

One of the primary study systems in my own laboratory is the comma butterfly *Polygonia c-album*. This species has a distinct seasonal polyphenism, linked tightly (but not absolutely) to diapause (Nylin, 1989, 1992). The directly developing summer morph has lightly coloured brown to ochreous undersides to the wing, whereas adults emerging late in summer and entering winter reproductive diapause before breeding in the spring have dark wing undersides coloured brown, black and green. The adaptive significance of this difference is not entirely clear, although it has been shown that the dark form is more cryptic and hence better protected from predators on tree trunks, probably a common background during hibernation (Wiklund & Tullberg, [Bibr b65]). The summer morph colour is possibly non-adaptive in itself, although more the result of allocation of nitrogen resources away from wing melanization, and towards a higher reproductive output (Karlsson & Wickman, 1989; Karlsson *et al*., 2008).

A very similar polyphenism also occurs in the two other Palearctic *Polygonia* species: *Polygonia c-aureum* and *Polygonia egea*. From the phylogeny of the genus, this appears to be the ancestral state, although the polyphenism has been lost twice as *Polygonia* colonized the New World. It can be speculated that this occurred during evolutionary phases when the populations were univoltine as a result of the likely northern route of colonization (Nylin *et al*., 2005). If this interpretation is correct, it provides indirect evidence for this polyphenism being upheld by selection in the Palearctic species. Traces of the ancestral polyphenism appear to remain in some Nearctic species because significant differences in adult colouration can be induced by increasing day lengths during the larval stage in *Polygonia satyrus* and *Polygonia gracilis*, although not in *Polygonia faunus*, the sister species to *P. c-album* (Nylin *et al*., 2005).

## Regulation of seasonal morphs

Below, two of the best studied systems regarding regulation of seasonal morphs are outlined. Other examples are provided in the recent review by Hartfelder & Emlen (2011).

### Aphid wing polyphenism

Aphids present a diversity of polymorphisms and polyphenisms, although most relevant in the context of the present review is the common ‘end-of-season switch’ from parthenogenetic generations during the summer to sexually reproducing forms in the autumn, which subsequently produce overwintering eggs (Simpson *et al*., 2011). The regulation of this switch has been studied in the host-alternating, black bean aphid *Aphis fabae*, in which the development of late-season winged females (induced by short days) that give birth to the wingless sexual females can be over-ridden by either long day lengths or high temperatures experienced in the first instar. These then develop into wingless adults, and their daughters are parthenogenetic, rather than sexual females. Such a scenario can be mimicked by topical application of JH (Hardie, 1980, 1981a, 1981b; Hardie & Lees, 1983). By contrast, the development of winged dispersal morphs, induced by nonseasonal cues such as crowding, is not coupled with a switch to sexuality, and does not appear to be regulated by JH (Hardie & Lees, 1985; Brisson, 2010; Hartfelder & Emlen, 2011).

The molecular basis of the formation of winged and wingless morphs is still largely unknown, although this may change because the genome of the pea aphid *Acyrthosiphon pisum* has recently been sequenced. One interesting piece of information comes from a comparison of gene expression in winged and wingless individuals of *A. pisum*, focusing on genes known to be involved in the wing formation in *Drosophila*. Brisson *et al*. (2010) report that the gene *apterous 1* is expressed differentially, and propose that it acts proximately to realize the wing polyphenism. In this case, it is a phenotype induced by crowding, suggesting a different upstream regulation from seasonal morphs (see above), although this part of the mechanism could still be shared (Brisson, 2010). Another gene of interest is *aphicarus*, which not only controls male wing production in *A. pisum* through a locus on the X-chromosome, but also affects the plastic propensity to produce winged forms under crowding, an illustration of the need to consider polymorphisms and polyphenisms together to make full use of the available information (Brisson, 2010).

### Melanism and eyespots in nymphalid butterflies

One of the best understood seasonal polyphenisms in terms of regulation is the seasonal variation in melanism, as commonly seen in nymphalid butterflies (the adaptive function is however less well understood, in contrast to the situation for pierid butterflies). In several species, melanism is regulated by photoperiod, as well as by temperature, independently but additively (Hartfelder & Emlen, 2011). A common mechanism, as exemplified by *Junonia coenia*, is that the dark form is the ‘default’, although there is a critical period of hormone sensitivity early in the pupal stage when the production of this form can be averted. Long days or high temperatures lead to early and high levels of PTTH secreted from the brain, in turn inducing above-threshold levels of ecdysteroids during the critical period, and production of the alternative light coloured phenotype (Rountree & Nijhout, 1995).

In *Polygonia* (studies made on the Asian *P. c-aureum*), there is evidence for a very similar induction mechanism but, instead of PTTH, a similar neurohormone, aptly named summer morph-producing hormone has been indicated. Pupae from larvae reared in long days form the default autumn morph if the brain has been dissected out, although injection of ecdysteroids before the critical period restores the summer morph (Endo *et al*., 1988).

The seasonal variation in eyespot presence and size seen in *Bicyclus* butterflies has been studied thoroughly and, interestingly, the mechanism of induction is similar to that for melanism, with a period sensitive to ecdysteroids during the first few days of the pupal stage (Koch *et al*., 1996). The molecular basis of the eyespot variation is also comparatively well known because butterfly eyespots have come to serve as something of a model system for control of pattern formation during development. Genes such as *distal-less*, *engrailed*, *spalt* and *wingless* have all been convincingly implicated in the circular pattern expression of eyespots (Beldade & Brakefield, 2002), and probably interact with ecdysteroids during the period when the pattern is formed (Koch *et al*., 2003).

## Knowns: a summary

The brief review provided above of some of the (more or less) known facts regarding the induction of diapause and seasonal morphs should suffice to demonstrate that there are many differences that can be observed: between diapause in different developmental stages; between diapause and seasonal morph regulation; and between taxa. At the same time, there are also many similarities, such as the induction by photoperiod and temperature; the important roles often seen of ecdysteroids, PTTH and JH, and the central role of the insulin signalling pathway. A simple unified model of induction may not be possible, even for diapause induction alone (Denlinger *et al*., 2011), although every effort should still be made to progress along such lines to allow broader generalizations.

## Unknowns: some things that are not fully understood

### The role of changing photoperiods

In *P. c-album*, there is evidence that responses to ‘critical photoperiods’ do not tell the whole story regarding the induction of reproductive diapause and seasonal morphs. In the Swedish population, which is normally univoltine, the dark (diapause) form is produced even in very long day lengths such as 18 or 20 h of light. However, if the day length is increased from 18 to 20 h during the larval stage, the majority of individuals develop into the light form and avert diapause (Nylin, 1989, 1992). In the English population, which is partially bivoltine in the field, there is a critical day length somewhere below 18 h, so that most individuals avert diapause in a constant 18 or 20 h light. However, if photoperiods decrease from 20 to 18 h during the larval stage, all individuals emerge as the dark form (Nylin, 1989).

These results indicate strongly that *P. c-album* is sensitive to the direction of change in photoperiod *per se*. It is not known how important and widespread such sensitivity is because, generally, only constant day lengths are used in the laboratory. When my own results for *P. c-album* were first published (Nylin, 1989), there were only a small number of previous studies that clearly showed such sensitivity. The speculation was put forward that the sensitivity to direction of change may be more likely to be found in species with adult diapause; such insects are likely to fly early in the spring, so that their offspring are subjected to the increasing photoperiods before summer solstice. This may present them with a problem of ambiguous information from ambient photoperiods: the same day lengths are present before and after summer solstice but, in many cases, only the former should induce direct development. Sensitivity to the direction of change (and not only critical day length) should resolve this dilemma. To my knowledge, this notion has never been followed up, and only a handful of new studies on sensitivity to changing day lengths have been published in the last two decades; for example, the study by Zhu & Tanaka (2004) regarding summer diapause in a subtropical cockroach.

### Sex differences and parental effects

Differences between the sexes in the propensity to enter diapause are found often, although the background and relevance of this is not at all clear. In butterflies, males generally enter the reproducing population before females (i.e. there is protandry, presumably as a result of selection on males to maximize their chances of mating with virgin females and on females to reduce the pre-reproductive period) (Wiklund & Solbreck, 1982). From this body of theory, it is hypothesized that males should be more prone to enter diapause at near-critical conditions for diapause induction because such late males are not likely to achieve protandry under direct development but, instead, could do so by breaking diapause early in the spring. A higher propensity of males to enter diapause is indeed found in *Pieris napi*, *Pararge aegeria* and *P. c-album* (Wiklund *et al*., 2004). Protandry after diapause has been studied in *P. napi*, where males are found to break diapause earlier than females under controlled conditions in the laboratory (Forsberg & Wiklund, 1988). From *P. aegeria* and *P. c-album*, such studies appear to be lacking.

In the monarch butterfly, *D. plexippus*, the sex ratio is skewed towards males at the overwintering sites, and different non-adaptive explanations such as a higher female mortality during migration have been proposed (Frey & Leong, 1993). Selection for protandry may be the ultimate explanation also for *D. plexippus* (i.e. the ‘missing’ females may not be dead but, instead, reproducing rather than migrating, to a greater extent than males) (Nylin *et al*., 1995); however, this suggestion has received little credit among researchers studying this species (Frey & Leong, 1995). In this context, it is of interest to note (see also above) that, when the monarch butterfly genome was published recently (Zhan *et al*., 2011), a sex difference in the molecular pathway of JH regulation was reported. Such sexual dimorphisms clearly indicate a potential for sex differences (e.g. in diapause propensity).

Crossing stock that originate from different locally adapted populations can give interesting insights into the genetic architecture behind such traits. The Spanish and Swedish populations of *P. c-album* have been reciprocally crossed, and the offspring reared under long-day conditions, resulting in almost 100% of the directly developing light form in the pure Spanish stock and a large majority of the dark form in Swedish stock (Söderlind & Nylin, 2011). The hybrids show rather intermediate frequencies but with male hybrids of both reciprocal crosses being more prone to enter diapause. Thus, the sex difference apparently over-rides any effects of the particular genetic backgrounds with their locally-adapted thresholds, again suggesting that the propensity for diapause may be a secondary sexual characteristic. Moreover, the hybrids differ significantly with respect to the direction of the cross, so that, in particular, male hybrids with Swedish fathers respond similar to pure-stock Swedish males, and not intermediately (Söderlind & Nylin, 2011).

The growing number of studies involving Lepidoptera that show a paternal effect similar to that in *P. c-album* [e.g. the cotton bollworm *Helicoverpa armigera* (Chen *et al*., 2012) and Asian corn borer *Ostrinia furnacalis* (Xia *et al*., 2012)] is intriguing. It suggests that the male parent has more influence on the incidence of diapause, although the precise mechanism and relevance is unknown. Because Lepidoptera males are homogametic, sex-linkage cannot readily explain such patterns. Parental effects had also been found previously in similar crosses between populations of *P. aegeria* from Sweden and Madeira (Nylin *et al*., 1994) but, in that study, female hybrids were more like the pure stock of their mothers, a pattern more compatible with sex-linkage.

### Temperature and diet

Whereas photoperiod is undoubtedly the most important environmental cue that regulates both diapause and production of seasonal morphs, the effects of temperature and diet in modifying this response are very common. The relevance is typically less clear. Are temperature and/or diet used as cues of future conditions, or do these environmental factors only affect the diapause induction indirectly and non-adaptively through their effects on metabolism and life history? There are three possibilities (Wedell *et al*., 1997; Dalin & Nylin, 2012):

1. Under naturally varying conditions, such as in the field, there may be a ‘trivial’ response very generally to low temperature and/or poor diet, in that the critical periods for photoperiodic induction may be delayed until the critical day length has been passed.
2. The poor conditions can also affect traits such as larval growth and phenotypic quality, which in turn somehow affect diapause propensity (also in constant photoperiods). Because it may be certainly non-adaptive to pursue direct development when conditions are poor, such responses could be strengthened and modified by selection in a process of genetic accommodation (West-Eberhard, 2003).
3. The external conditions can even be selected to function as environmental cues *per se*, indirectly of their effects on other traits. They could be cues of seasonal progress, or perhaps more generally as cues of future prospects for growth and reproduction in the same season.

Variation in temperature clearly has strong potential to affect the life cycles of ectotherms non-adaptively in various ways, although there are some cases where an adaptive function is clearer. The seasonal polyphenism in *B. anynana* eyespots is regulated by temperature (and diet) through the effect on development time (Brakefield *et al*., 1998), apparently with no effect of photoperiod. In a very interesting study of the regulation of adult seasonal melanization in *Colias* butterflies, Hoffmann (1978) shows that the relative roles of temperature and photoperiod vary with their ability to predict future environmental conditions. Photoperiod is typically used as a cue for both diapause and seasonal morphs because it is a ‘noise-free’ signal of seasonal progression so that, although seasonal variation in melanization is considered to be an adaptation to regulate temperature, it is not actually induced by temperature. However, in some populations, photoperiod is a poor predictor of future temperatures as a result of local climatic conditions, and, here, photoperiod regulates diapause but not seasonal morph, which is instead affected directly by the temperature during the pupal stage. Unfortunately, few attempts have been made to pursue this promising line of investigation in this system or in other insects.

Returning to *P. c-album*, both temperature and diet (larval host plant) affect diapause propensity strongly, in addition to photoperiod (the main cue) and sex (Wedell *et al*., 1997). The effect of temperature may or may not be adaptive by itself because high temperatures to some extent signal summer conditions but are a relatively poor cue of seasonal progression. Regarding dietary effects, it is found that the effect on diapause propensity exactly matches the preference ranking of ovipositing females, which in turn matches the growth rate that larvae can accomplish on different plants, with the preferred host nettle (*Urtica dioica*) resulting in the highest percentage of the light, directly developing, form, and the slow-growth host birch (*Betula pubescens)* resulting in the the lowest. Host plants may be used as a true cue of future growth prospects by *P. c-album* (the third possibility above), although the evidence is only a correlation so far (the effect remains after controlling for larval growth rates, partly ruling out the second possibility).

The leaf beetle *Phratora vulgatissima* is another species that appears to respond adaptively to host-plant quality by the induction of adult diapause (Dalin & Nylin, 2012). Both larvae and adults of this species feed on willows (*Salix* spp.), including field species such as *Salix phylicifolia*, as well as plantations of *Salix viminalis*. Because the latter is harvested for energy production, there is more re-growth and higher quality food compared with field species of *Salix*, and plantations are also where the partial second generation of *P. vulgatissima* is most commonly seen. The ‘trivial’ explanation (i.e. the first possibility) for this pattern can be ruled out because adults actually emerge at the same time. For the same reason, an effect through larval growth rates (i.e. the most obvious version of the second possibility) can also be ruled out. Instead the critical photoperiod is itself affected by food quality, and individuals reared on high quality food (with lower diapause propensity) have higher quality in terms of fat reserves. This suggests either another adaptive version of the second possibility, in which direct development is favoured when there are sufficient fat reserves for immediate reproduction, or that the chemical properties of the plant are indeed used as a direct cue of prospects for growth and reproduction by the beetles.

## Integration: some open questions

A model of diapause regulation as a series of modules is outlined above, in accordance with Emerson *et al*. (2009). Some limitations to this model (for the purposes of the present review, which has a broader scope than the original publication) can now be noted, representing further challenges to the full understanding of the regulation of diapause and seasonal morphs:

1. There is no mention of how the direction of change in photoperiod is detected by insects that are sensitive to such information from the environment (or are they all sensitive, even though only a few species clearly respond?). It can be assumed however, that the ‘photoperiodism module’ is responsible.
2. The only environmental cue included in the model is photoperiod, although there are clearly cases in which other cues are also of importance; and even cases when photoperiod is not involved. It is not at all clear how other environmental factors (whether they act as true cues or not) exactly affect the modules. Because temperature has such ubiquitous effects on ectotherms, it may affect any or all of the modules, including the upstream ‘photoperiodism module’. The effect of diet is likely to act more downstream, perhaps by modifying the output of the ‘hormonal events’ module. Diet could also affect the ‘diapause module’ directly, when it is used as a true cue (the third possibility) but, because the co-adaptation between diapause and seasonal morph is typically preserved even when the diet varies, this is less likely.
3. Seasonal morphs and other polyphenisms are not included in the model. A particular polyphenism, which is not simply part of the core diapause syndrome, can be considered as an additional module. There are two possibilities for how such a module could be added to the model of Emerson *et al*. (2009): either as an alternative recipient of output from the ‘hormonal events’ module or downstream of the ‘diapause module’. The latter may be the case for some polyphenisms that are actually direct effects of the diapause (and as such potentially non-adaptive, no matter how striking they are). However, the physiology and genetics of diapause and seasonal morphs are often at least partially independent of each other (e.g. in *P. c-album*; Nylin, 1992), suggesting that the former possibility is more general. If this is the case, the fact that the two traits often occur together is a result of co-adaptation, where the shared ‘selective regimes’ have lead the two traits to convergence on similar seasonal cues and sometimes on the same regulating hormones.

## Conclusions

Some ‘key players’ in the regulation of diapause and seasonal morphs have been identified, in the form of seasonal cues (in particular photoperiod), hormones (in particular PTTH, ecdysteroids, JH) and the insulin signalling pathway. Furthermore, the molecular basis for the regulation is starting to emerge. However, there is not yet a full understanding of how seasonally occurring traits are integrated with each other to form a co-adapted complex phenotype, which is adaptive in the wild and responds adaptively to environmental and organismal variation: photoperiod change, temperature, diet, sex, parental origin and other factors not covered in the present review.

The only way to accomplish this is through integrative biology, where ecologists, physiologists and molecular biologists come together to resolve these issues. A major challenge for such efforts is the problematic gap that exists between laboratory model organisms such as *D. melanogaster*, for which fantastic molecular tools exist, and the ecological model organisms, such as butterflies, for which there is a wealth of understanding regarding adaptation in the wild. If this gap can be bridged, the genomics revolution will provide an unparalleled opportunity for producing detailed, synthetic, mechanistic models of how cues and physiological pathways are interconnected via gene action, and perhaps even for understanding gene action under field conditions, rather than only in the shielded environment of the laboratory.
